# Micro-Segregated Liquid Crystal Haze Films for Photovoltaic Applications: A Novel Strategy to Fabricate Haze Films Employing Liquid Crystal Technology

**DOI:** 10.3390/ma11112188

**Published:** 2018-11-05

**Authors:** Jae-Hyun Bae, Eui Dae Jung, Yun Seok Nam, Byeong-Cheon Kim, Hyeon-Joon Choi, Hyun Gi Kim, Myoung Hoon Song, Suk-Won Choi

**Affiliations:** 1 Department of Advanced Materials Engineering for Information and Electronics, Kyung Hee University, Yongin 17104, Korea; scr4706@naver.com (J.-H.B.); rlaqudcjs15@naver.com (B.-C.K.); legend930130@gmail.com (H.-J.C.); 2 School of Materials Science Engineering, Ulsan National Institute of Science and Technology, Ulsan 44919, Korea; jed9318@unist.ac.kr (E.D.J.); namyunsuk7@unist.ac.kr (Y.S.N.); mhsong@unist.ac.kr (M.H.S.); 3 Regional Innovation Center-Components and Materials for Information Display, Kyung Hee University, Yongin 17104, Korea; opti_people@khu.ac.kr

**Keywords:** optical haze, liquid crystal, photovoltaic, polymer

## Abstract

Herein, a novel strategy to fabricate haze films employing liquid crystal (LC) technology for photovoltaic (PV) applications is reported. We fabricated a high optical haze film composed of low-molecular LCs and polymer and applied the film to improve the energy conversion efficiency of PV module. The technique utilized to fabricate our haze film is based on spontaneous polymerization-induced phase separation between LCs and polymers. With optimized fabrication conditions, the haze film exhibited an optical haze value over 95% at 550 nm. By simply attaching our haze film onto the front surface of a silicon-based PV module, an overall average enhancement of 2.8% in power conversion efficiency was achieved in comparison with a PV module without our haze film.

## 1. Introduction

Environmentally friendly energy generation devices such as photovoltaic (PV) cells have attracted much attention because of the depletion of fossil fuels as well as the environmental issues arising from the use of these fuels. Hence, the development of high-performance PV cell technologies is a pressing global need, and many attempts have been made to improve power conversion efficiency (PCE) at low cost [1,2,3,4,5,6]. Among these, the integration of optical thin films, which manipulate the behavior of incident light by inducing antireflection [7,8,9], resonance [10,11], and scattering [12,13], with PV cells has gained attention as a promising method for coupling as much light as possible into the cells [14]. Ideally, these light management films should neither exhibit parasitic absorption nor cause a deterioration in cell performance [15]. Recently, haze thin films have been actively employed as a light management layer in optoelectronic devices to enhance the absorption efficiency of PV cells [6,15,16,17].

Herein, our novel strategy for combining liquid crystal (LC) technology with a PV cell is proposed. Self-aggregating and self-assembling properties of LCs are very useful for developing novel functional devices [18,19,20]. We explore new haze films that apply LC technology for PV applications. Optical haze can be used to manipulate light behavior, and is given by:

Optical haze = DT/TT × 100(1)

where DT is the diffusely transmitted light, and TT is the total transmitted light [21].

In this work, we demonstrate PCE enhancement of a PV module using a LC technology-based optical film that exhibits a high haze performance, which is caused by the diffusion of light associated with low molecular micro-segregated LCs (MSLCs) surrounded by a polymer medium. This MSLC is made using a simple mixed system of low molecular LCs and polymers. In general, a polymer-dispersed LC (PDLC) consists of LC droplets dispersed in a polymer matrix. A PDLC can be electrically switched from a light-scattering (opaque) state to a non-scattering (transparent) state for smart windows or display applications [22,23,24]. However, in this work, we only focus on the optical haze properties of a spontaneously phase segregated film with LC droplets a few micrometers in size embedded in a polymer medium for PV applications without electrical switching. Hence, our fabricated film is called MSLC instead of PDLC to highlight the functional differences between the two films.

Our MSLC film elongates the optical path length of light and hence the light absorption in the active layer of the PV module is boosted through diffusion of the incident light [15,25]. We optimized the optical performance of our haze film by adjusting UV curing temperatures, and the mixing ratio of LC and polymer. Under optimized conditions, the MSLC exhibited a high optical haze value up to 97% (at 550 nm). By simply attaching this MSLC film onto the front surface of a silicon-based PV module, we achieved an overall PCE enhancement of ~2.8% in comparison with a bare PV module.

## 2. Materials and Methods 

**Figure 1:**
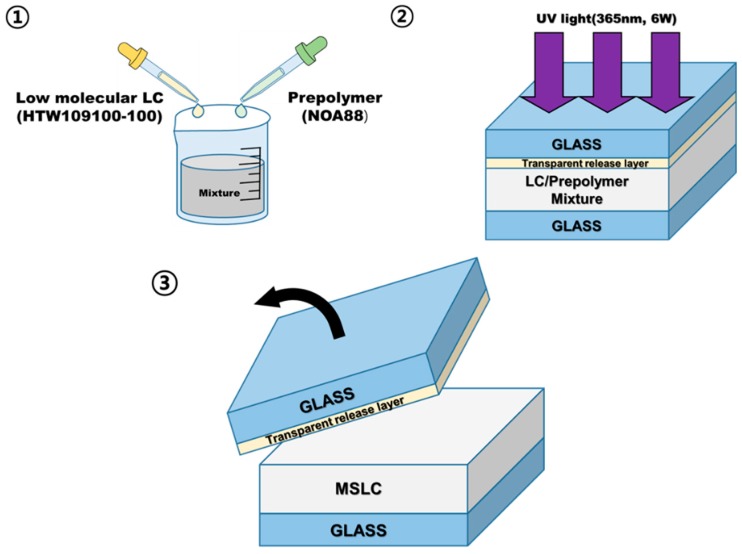
Schematic illustration of the fabrication procedure for our micro-segregated liquid crystal (MSLC) film.

We fabricated a MSLC haze film composed of LC/polymer composite with haze properties by employing a spontaneous phase separation process between low molecular LC and a polymer medium [26,27]. Figure 1 is a schematic of the procedure used to fabricate the MSLC film. First, a homogeneous mixture consisting of LC and photo-curable prepolymer was prepared. The prepared mixture was inserted by capillary action into asymmetrical cells with two glass substrates treated to achieve different surface conditions. The surface of upper substrate was coated with a transparent release layer. In contrast, no surface treatment was performed on the lower substrate. Film thicknesses were fixed using 30 μm glass beads as spacers. Then, the cell filled with the homogeneous mixture was placed on a hot stage to adjust the curing temperature, and exposed to UV light (365 nm, 6 W) for 30 min. Once the polymerization reaction of the blended prepolymer was initiated in the homogeneous mixture, spontaneous polymerization-induced phase separation occurred between the LCs and polymers. As a result, LCs came out of the homogenous phase and began to form LC droplets. The droplets grew until the prepolymer became sufficiently solid to trap the LCs and prevent them from moving easily [28]. The LC droplet size varied based on the curing temperature and mixing ratio of materials used, and was typically on the order of a few micrometers to several tens of micrometers. Finally, the release layer-coated glass substrate was removed at room temperature (RT), which yielded a MSLC thin film on a glass substrate.

To fabricate MSLC film, commercially available low molecular LC (HTW109100-100, HCCH) and photo-curable prepolymer (NOA88, Norland) were employed. Extraordinary refractive index of the LC used here is *n_e_* = 1.706 (at 20 °C, 589 nm), and refractive index of the prepolymer is *n* = 1.56. 

The TT and DT spectra of the haze films were measured using a UV-Vis-NIR spectrophotometer (Cary 5000, Agilent) with an integrating sphere (inner diameter: 60 mm). Optical haze was calculated using Equation (1). The bidirectional transmittance distribution function (BTDF) is used when specifically referring to transmitted scatter through a medium as a function of the angular positions of the incident and scattered beams. The BTDF was evaluated using SDR-300S (J&C Technology Co., Ltd., Busan, Korea).

The J–V curves of the PV cells were obtained using a Keithley model 2400 source measurement unit under AM1.5G simulated illumination (100 mW cm^−2^). External quantum efficiency (EQE) measurements were obtained as functions of wavelength with incident photon-to-current conversion equipment (PV Measurements Inc., Point Roberts, WA, USA).

## 3. Results and Discussion

**Figure 2:**
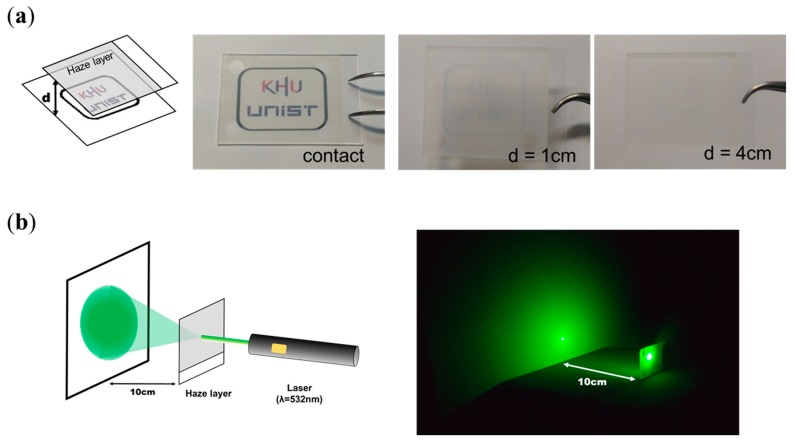
(**a**) Photographs of the MSLC film placed between the printed logo and the camera. The MSLC film is spaced apart from the logo by varying distances (d = 0, 1, and 4 cm). (**b**) Image of a green laser (λ = 532 nm) beam irradiated onto the screen after passing through the MSLC film.

Firstly, preliminary tests of our fabricated MSLC film were performed. Figure 2a shows photographs of the MSLC film placed between the printed logo and the camera. When the film was placed on top of the logo, a clear image was observed. This demonstrates the high optical transparency of our MSLC film. When the film was lifted off the logo, a cloudy image was observed because the light transmitted via the film became highly diffused. We also took an image of a green laser (λ = 532 nm) beam irradiated onto the screen after passing through the MSLC film. As shown in Figure 2b, the laser beam is highly diffused after passing through the MSLC film. Thus, the feasibility of using the fabricated MSLC film as a haze film was confirmed.

**Figure 3:**
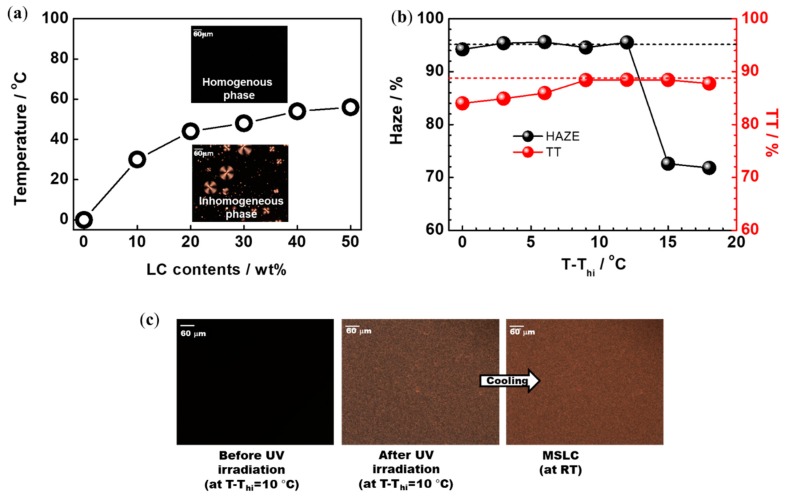
(**a**) Phase diagram of the liquid crystal (LC)/prepolymer mixtures with different mixing ratios. Typical polarized optical microscopy (POM) images of a LC/prepolymer mixture (30:70 wt%) above and below T_hi_ are also shown in the inset. (**b**) Optical haze and total transmitted (TT) (at 550 nm) of an MSLC film with 30 wt% LC at various UV irradiation temperatures. (**c**) POM images of the MSLC film with 30 wt% LC before and after UV irradiation at a temperature 10 °C higher than T_hi_.

Typical polarized optical microscopy (POM) images of a LC/prepolymer mixture (30:70 wt%) before UV irradiation are shown in the inset to Figure 3a. As shown in the figure, the homogenous fluid phase changed to a relatively inhomogeneous phase with the unintended formation of LC droplets without UV irradiation upon cooling at a rate of 1 °C/min. We identified the transition temperature (T_hi_) from the homogenous phase to the inhomogeneous phase on cooling, and plotted a phase diagram of the LC/prepolymer mixtures with different mixing ratios, as shown in Figure 3a. In order to induce polymerization-induced phase separation between LCs and polymers in the homogeneous phase, UV irradiation was performed above T_hi_. Figure 3b depicts the optical haze and TT (at 550 nm) of an MSLC film with 30 wt% LC at various UV irradiation temperatures. The haze and TT of the MSLC film were evaluated at RT. More than 95% haze and around 89% TT were exhibited when the films were cured ~10 °C above the T_hi_. Figure 3c shows POM images of the MSLC film with 30 wt% LC before and after UV irradiation at a temperature 10 °C higher than T_hi_. By means of UV irradiation, LCs came out from the homogeneous phase and formed LC droplets a few micrometers in size. The spontaneous phase segregated state, MSLC, was kept at RT because the prepolymer solidified such that the LCs were trapped. When light passed through the MSLC film, these micro-LC droplets acted as light scattering centers due to the refractive index-mismatch between the LCs and the polymer [29].

**Figure 4:**
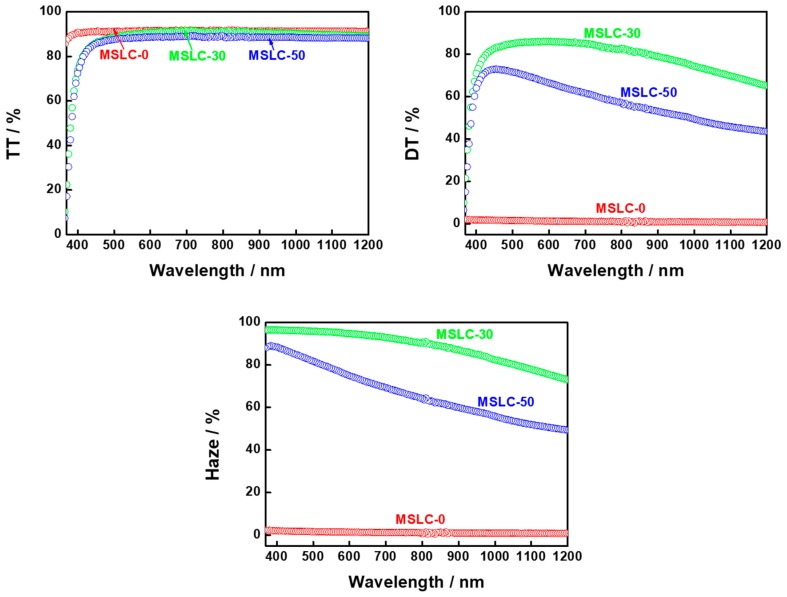
TT, diffusely transmitted (DT) and the optical haze spectra of three MSLC films using different LC mixing ratios at room temperature (RT).

Figure 4 shows TT, DT and the optical haze spectra of three MSLC films using different LC mixing ratios at RT. Each MSLC film had noticeably different haze, which was dominantly affected by the LC mixing ratio. MSLC-0 consisted of 100 wt% polymer film without LCs and was UV irradiated at RT. (Strictly speaking, MSLC-0 is not MSLC film.) The LC mixing ratios for MSLC-30 and MSLC-50 were, respectively, 30 wt% and 50 wt%. UV irradiation for both MSLC-30 and MSLC-50 was performed at ~10 °C above the T_hi_. 

**Figure 5:**
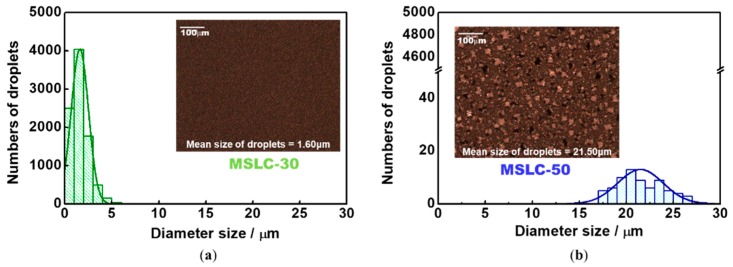
Histograms of the LC droplet size distribution in the MSLC-30 and MSLC-50 films, respectively. Typical POM images of the two films are also provided in the insets.

Figure 5 shows histograms of the LC droplet size distribution in the MSLC-30 and MSLC-50 films, respectively. Typical POM images of the two films are also provided in the insets to Figure 4. Although the TT levels of the two films were similar, the DT levels were distinctly different for the two films; MSLC-30 exhibited a larger DT than MSLC-50. It was revealed that the LC droplet size is the main factor affecting the DT of the films; MSLC-30, which had smaller droplets, showed a relatively higher DT. Because the MSLC-30 had smaller LC droplets, it had more numerous scattering centers, and therefore exhibited a higher DT than the MSLC-50 film, which had larger LC droplets. Consequently, the MSLC-30 film exhibited a high optical haze value around 97% (at 550 nm). MSLC-10 and MSLC-20 with 10 and 20 wt% LC contents, respectively, were also fabricated and evaluated. The haze properties MSLC-20 exhibited were similar with those of MSLC-30. On the other hand, the optical haze was decreased in MSLC-10. This is because although LC droplet size was decreased, scattering centers also decreased by means of the small amount of LC contents.

We found that the LC droplet size is an important parameter influencing the optical haze in our films. It was revealed that the MSLC film with small LC droplets (a few micrometers) exhibited a higher haze value than the film with large LC droplets (a few tens of micrometers). In the case of PDLC for switchable smart windows (displays), small LC droplets are unfavorable because they increase the driving voltage [24,30]. A small droplet size enhances the total area of the interface between the LCs and the polymer, and the LC molecules on the polymer surfaces are strongly anchored. Consequently, a higher electric field is required to move the LC directors in the droplet. In contrast, the small droplet size in the MSLC films was favorable from the viewpoint of haze as a functional optical film.

**Figure 6:**
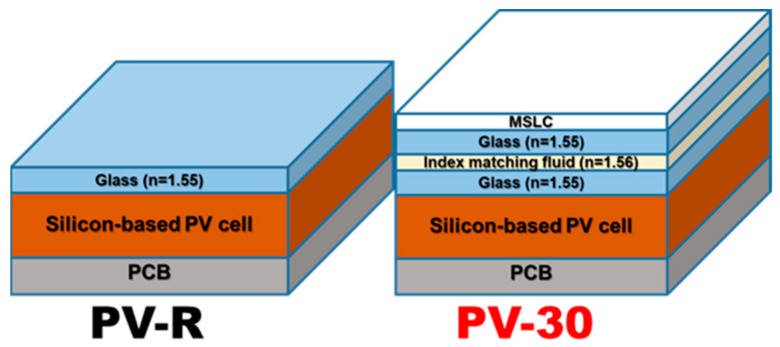
Schematic illustration of the two modules evaluated in this work.

**Figure 7:**
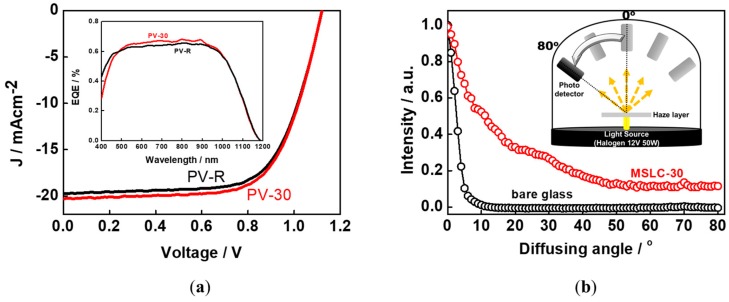
(**a**) Current-voltage (J–V) curves of PV-R and PV-30. External quantum efficiency (EQE) curves of the two modules are also presented in the inset. (**b**) Bidirectional transmittance distribution function (BTDF) of transmitted light through MSLC-30 and bare glass. A schematic diagram of the measurement set-up for BTDF is also provided in the inset. Light that was vertically incident on the film was used as the incident beam.

To demonstrate the applicability of the MSLC film with optical haze behavior in a PV module, we integrated the film with a commercially available silicon-based PV module (itemSchool). To investigate the performance improvement caused by the integration of the MSLC film, we prepared two modules; PV-R and PV-30. Schematic illustrations of the two modules are depicted in Figure 6. PV-R, encapsulated on a printed circuit board (PCB) together with a glass cover-substrate (refractive index *n* = 1.55), was the reference module without the MSLC film. PV-30 was a silicon-based PV module that had MSLC-30 film integrated onto its front surface via refractive index matching fluid (*n* = 1.56). The PV performance of PV-30 was investigated against that of PV-R using a solar simulator under AM 1.5 G conditions (1000 Wm^−2^). The typical current-voltage (J–V) curves are presented in Figure 5b. The open circuit voltage (*V_oc_*), short circuit current density (*J_sc_*), fill factor (FF), and power conversion efficiency (PCE) of the two PV modules are summarized in Table 1. The data represent the average of 15 measurements. By attaching our MSLC film onto the front surface of a PV module, an average PCE enhancement of 2.8% was achieved in comparison with a PV-R. During 15 measurements, PV-30 exhibited a maximum (minimum) PCE enhancement of 3.7% (1.2%) compared to that of PV-R. Thus, the PCE enhancement was reliable. As shown in Table 1, the PCE enhancement is only owing to an increase in the *J_sc_*. The EQE curves of the two modules are also presented in the inset to Figure 7a. Enhanced EQE is observed over a wide wavelength range, from around 500 nm to 1000 nm. Theoretically calculated *J_sc_* values of the two PV modules also showed similar trends as the experimentally evaluated ones. Since the only difference between the two PV modules was in the attached film, the higher EQE values for the MSLC-attached PV cells indicate enhanced harvesting of the incident solar light due to the haze behavior of the MSLC films. 

To explain PCE enhancement of the PV-30 compared to that of the PV-R, we investigated the BTDF of transmitted light through MSLC-30 and bare glass. As presented in Figure 7b, normally incident light is diffused with oblique angles after it passes through MSLC-30. In other words, the strong diffusion of incident light passing through the MSLC film changed the propagation direction of the light from normal to oblique incidence in the PV cell. Consequently, the optical path length of the light was elongated and hence the light coupling into the active layer was also enhanced by our MSLC film. However, parasitic losses (such as the absorption of light by the MSLC film and total internal reflection at the interface between the MSLC film and PV module) in our PV-30 may limit the overall enhancements in device performance. Nevertheless, the PCE of PV-30 was superior to that of PV-R. Thus, greater PCE enhancements would be observed if the above parasitic losses were suppressed.

The haze properties MSLC-20 exhibited were similar to those of MSLC-30. Thus, the PCE enhancement that the PV module with MSLC-20 showed was similar to that of PV-30. On the other hand, the PV module with MSLC-10 (MSLC-50) was inferior to the PV module without MSLC films because the optical haze was decreased in MSLC-10 (MSLC-50).

## 4. Conclusions

We evaluated haze films using LC technology for PV applications. A new kind of high-performance haze film, namely the MSLC film, which improves the PCE of PV cells is reported herein. Our MSLC film provided a simple, effective, and low-cost method of further enhancing PV cell efficiency employing LC technology. MSLC film was made from a simple mixed system of low molecular LCs and polymers, and fabricated by polymerization-induced phase separation between the LCs and polymers. We optimized the optical performance of our haze film by adjusting the mixing ratio of LC and polymer, and UV curing temperatures. It was revealed that the MSLC film with small LC droplets (a few micrometers) showed higher haze value than that with large LC droplets (a few tens of micrometers). Under optimal conditions, the MSLC exhibited a high optical haze value up to ~97% (at 550 nm). By simply attaching this MSLC film onto the front surface of a silicon-based PV modules, an overall PCE enhancement of 2.8% in comparison with the bare PV module was achieved. The improvement in the PV performance was attributed to enhancement of the light absorption in the active layer of the PV cell through diffusion of the incident light via the haze film. Our results demonstrate the promising potential of MSLC films, which can be easily fabricated using simple and inexpensive LC technology for all types of PV devices. We expect that our approach will be widely used for developing high-performance and cost-effective PV devices in the future.

## Figures and Tables

**Table 1:** Measured J-V characteristics of PV-R and PV-30. Data represent the average of 15 measurements. ↑ The arrow represents the percentage increase of PCE compared to the PCE of the PV-R.

Module	*J_sc_* (mA/cm^2^)	*V_oc_* (V)	*FF* (%)	*PCE* (%)	*PCE* ↑ (%)
PV-R	19.73	1.12	67.37	14.90	
PV-30	20.30	1.12	67.37	15.32	2.8
